# Medicine and Horsemanship: The Effects of Equine-assisted Activities and Therapies on Stress and Depression in Medical Students

**DOI:** 10.7759/cureus.6896

**Published:** 2020-02-05

**Authors:** Pressley A Chakales, Jacklyn Locklear, Tracy Wharton

**Affiliations:** 1 Neurology, University of Central Florida, Orlando, USA; 2 Obstetrics and Gynecology, University of Central Florida, Orlando, USA; 3 Internal Medicine, University of Central Florida, Orlando, USA

**Keywords:** stress, depression, equine assisted, animal assisted, medical students, mental health

## Abstract

This study examined the use of an equine-assisted brief course module on stress and depression among medical students (n = 28), a demographic known to experience high pressure. Evidence supports that animal-assisted therapies can lead to the improvement of health and quality of life, particularly in terms of cognitive, psychological, and physical benefits. This study used the seven-session Kane Medicine and Horsemanship program; students completed pre- and post-measures one week before and after the course. Participation in the course significantly reduced perceived stress (p: 0.001), depression (p: <0.001), stress severity (p: 0.014), and stress frequency (p: 0.001) among medical students. This approach should be further investigated as an option for improving well-being among medical students.

## Introduction

According to the World Health Organization (WHO), depression is a principal cause of disease burden worldwide [1]. Evidence supports strong linkages between depression and chronic health burden, as well as suicide completion. One particularly vulnerable subgroup for depression is medical students. Substantial evidence supports the existence of significant levels of stress and depression among medical students, which are 2-5 times higher than in the general population [2,3]. A meta-analysis by Rotenstein et al. indicated that these levels of stress and depression in medical students are on the rise, along with suicidal ideation. The review analyzed 195 studies involving 129,123 medical students worldwide and found that more than a quarter of the sample population screened positive for depression and more than one in ten indicated suicidal ideation while in medical school [3]. This finding is a cause for extreme concern and immediate measures should be implemented to find solutions to relieve student depression, stress, and other factors that may lead to suicidal ideation, as well as other health issues such as sleep and eating disturbances, cardiac strain, and high-risk behaviors.

Although research has shown that all college students are at risk for high rates of stress, there is something unique about medical school [4]. A longitudinal study of students' depression conducted at one medical school reports that while the rate of depression in students beginning medical school is similar to other people of the same age, the prevalence of depression in medical students increases disproportionately during the course of their training [5]. Data also showed that the increased depression rates persisted over time as a chronic, rather than episodic, issue [5]. The cumulative stress model in a study by Lazarus suggests that the continued pressure of training programs would lead to increased stress as time goes on, but there is some evidence that coping strategies may mitigate distress and that rates of depression may reduce by year five if students are able to put such coping strategies to use [4,6-8]. Many medical schools are searching for ways to promote student health and wellness, and the identification and implementation of programs and initiatives to achieve these are necessary to ensure the health and well-being of our future physicians. 

Animal-assisted therapy

Animal-assisted therapy, especially equine-assisted activities and therapy (EAAT), has been used as a strategy to improve health conditions and quality of life for patients with chronic conditions and to assist individuals to develop coping skills [9,10]. Evidence suggests that EAAT and the formation of the human-animal bond that occurs during the sessions can produce cognitive, psychological, and cardiovascular benefits [11]. Best known as an intervention popular among military veterans, equine-facilitated intervention is grounded in the concept of extra sensitivity of prey animals, compared to other animals such as cats and dogs, to affective congruity, body language, and expressed emotion, which is thought to be a useful therapy tool [12,13].

Research on EAAT models has shown a significant reduction in stress symptoms, symptoms secondary to trauma, and a significant increase in mindfulness skill use among individuals experiencing posttraumatic stress [14-16]. EAAT has shown promise as an approach that may have a positive impact on many demographics. Further research into its use with populations who experience sustained stress, such as medical students, can provide new insight into opportunities to impact outcomes for these populations [9,11,17,18].

Medicine and horsemanship 

Medicine and Horsemanship (M&H) is based on a course originally designed by a physician from Stanford University. It is currently taught as an elective to medical students at Stanford [19]. The goal of the course is to help students develop interpersonal skills, leadership qualities, and self-care techniques; the interaction with the horses provides an opportunity for stress reduction and reflection as well, which is consistent with EAAT theory. The format of the course promotes the development of interpersonal communication skills and teamwork among the students, which are critical transferable skills in medicine. Using the M&H course model, Goldberg et al. found that participation in the course led to significantly improved levels of nonreactivity and decentering aspects of mindfulness [15]. Given high rates of stress and depression among medical students, it is hypothesized that engagement in the course will reduce scores on validated measures of these constructs.

This study used Kane’s The Manual of Medicine and Horsemanship to structure the seven-session course, which was offered as an elective to first- and second-year medical students at the University of Central Florida, Orlando, FL [20]. The M&H program offers participants the opportunity to familiarize themselves with horse behavior and reflect on experiences with the horses and group members. Each session consists of a pre-brief involving an overview of the day’s session, a three-hour horsemanship activity, and a session debrief that focuses on developing insights into self-regulation of emotion, mindfulness, and behavior (both horse and human). The debrief sessions, self-guided by the medical students, also give participants the opportunity to share their experience and incorporate feedback for future sessions.

## Materials and methods

Study design 

This was an observational cohort study of enrolled four-year medical students. All students in each medical-school year cohort were offered the opportunity to register for the course as an elective module. Participants in the course were given pre- and post-assessments online and were asked for permission to use their data in research. Data were collected online through the Qualtrics system (Qualtrics, Provo, UT). Since students logged into this system through the university, their responses could be matched for pre- and post-assessments. Once data were exported into Statistical Package for the Social Sciences (SPSS) software (IBM, Armonk, NY), codes were assigned to participants and data were deidentified.

The course used the same faculty facilitator and supporting equine staff each time, and they were guided by a program manual. Seven sessions were held over a span of two months during the academic calendar for approximately three to four hours each at a local Professional Association of Therapeutic Horsemanship International (PATH Intl.)-certified therapeutic riding center. Participants completed the pre-assessments one week prior to and the post-assessments one week after the course. The measures consisted of the Beck Depression Inventory-II (BDI-II), Evaluation of Stress Factors (ESF) adapted scale, and the Perceived Stress Scale (PSS). Participants were given 10 dollars each as an honorarium for their time after completing pre-assessments and again after completing post-assessments. This study was reviewed and approved by the University of Central Florida Institutional Review Board (SBE-14-10228).

Sample

Participants were medical students enrolled in the first year or second year of medical school at the University of Central Florida. There was a total of 28 participants. Among the men (n = 5), two had no previous experience with horses and one had extensive experience. Among the women (n = 23), 10 had no previous experience with horses and three had extensive experience. All students who began the class completed it, and all agreed to the use of their de-identified data.

Measures

*Beck Depression Inventory-II* *(BDI-II) *[21,22]

The BDI-II is a 21-item self-report measure of the severity of cognitive-affective and somatic depressive symptoms. Items are scored on a four-point scale, ranging from 0 to 3. Scores are summed up across items for a total score ranging from 0-63, with high scores reflecting greater severity. The BDI-II has a sensitivity of 81%, a specificity of 92%, and α-levels ranging from 0.73 to 0.96 [23,24]. 

*Evaluation of Stress Factors* *(ESF)* [25] 

The ESF was adapted from the original study, to make it more appropriate to an American medical school and due to concerns about length. The original scale included 29 items, many of which identified issues contextual to the Nepalese origin of the study, such as “difficulty in the journey back home,” “living conditions in hostel,” “political situation of the country,” and “non-availability of adequate learning materials.” Twelve items were selected from this scale that captured the major issues identified in the literature; these were: performance in exams, academic curriculum, lack of time for recreation, competition with peers, performance in practicals, loneliness, relationships with the opposite sex, difficulty reading textbooks, adjustment with roommates, sleeping difficulty, exercise, and alcohol/drug use/smoking.

The ESF has two subscales, measuring stress severity for each item on a Likert scale of 1-10, with 1 being not severe and 10 being most severe, and stress frequency, rated from 1-5, as never, rarely, sometimes, often, and always. Scores are summed up across items for total scores ranging from 1-120 (stress severity) and 0-40 (stress frequency), with higher scores indicating an increased perception of stress factor severity and frequency. Because the scale was altered to better fit the target population, benchmarks were not used for cutoff scores, and the measure was used solely to create a baseline for comparison within the group.

*Perceived Stress Scale* *(PSS)* [7,26]

The PSS measures global stress over the previous month, with queries like “In the last month, how often have you felt confident about your ability to handle your personal problems?” Items are scored on a 5-point Likert scale from never (0) to very often (4). Scores are reverse coded for four questions and summed for a total score ranging from 0-40, with high scores indicating higher perceived stress. The reliability of the PSS ranges from Cronbach α of 0.85 to 0.91 with a test-retest reliability of 0.85 [27-29].

Data analysis

Due to the small sample size, a Wilcoxon two-sample rank-sum test was used to analyze the PSS and BDI. The ESP was examined by using the summed-up scores for both frequency and severity. Individual factors were also compared by pre- and post-assessment, using paired sample t-testing (with an alpha level of 0.05), and were also run using the Wilcoxon signed-rank test for verification.

## Results

Using sum totals of the PSS for pre- and post-assessment (n = 28; mean 1 = 20.61; mean 2 = 15.46), there was a significant difference found (p: 0.001). Similarly, for the BDI, there was a significant difference (p: <0.001) at pre- (mean 1 = 10.68) and post-assessment (mean 2 = 4.64). For the BDI, benchmarks for clinically significant depression were: 0-13 = minimal depression, 14-19 = mild depression, 20-28 = moderate depression, and 29-63 = severe depression. Three participants met the criteria for mild depression at baseline, two met the criteria for moderate depression, and one met the criteria for severe depression. At post-assessment, only two of these seven participants met the criteria for mild depression, while the remainder had dropped below clinical criteria. Of the participants who had scored in the sub-clinical range at baseline (10-13; n = 7), five had dropped to scores of one or three, one remained at the same score of 10, and one had a score that increased to the lower end of the mild depression range (15).

For the ESF, the difference between overall pre- and post-stressor frequency and severity scores were found to be statistically significant (p: 0.001 for frequency; p: 0.014 for severity). However, on closer examination, most of the individual factors showed no significant differences pre and post. Movement on the scale was seen for the frequency factors (using a 0-4 scale) of loneliness (mean 1 = 2.86; mean 2 = 2.32; p: 0.007), relationships with the opposite sex (mean 1 = 2.86; mean 2 = 2.39; p: 0.013), sleeping difficulty (mean 1 = 2.71; mean 2 = 2.32; p: 0.025), and exercise (mean 1 = 2.82; mean 2 = 2.39; p: 0.02). For severity (using a 10-point scale), the only significant differences were for the factors of academic curriculum (mean 1 = 6.04; mean 2 = 5.29; p: 0.055), difficulty reading textbooks (mean 1 = 3.21; mean 2 = 2.46; p: 0.020), sleeping difficulty (mean 1 = 4.46; mean 2 = 3.79; p: 0.039), and exercise (mean 1 = 4.36; mean 2 = 3.50; p: 0.030). Of the severity scores, only academic curriculum rose above the middle of the scale; so severity overall was generally low, while frequencies for statistically significant findings were above the median score on the scale (Table 1).

**Table 1:** ESF - factor means and significance Frequency is measured on a scale of 0-4 (higher score = greater frequency); severity is measured on a scale of 1-10 (higher score = greater severity) *Statistically significant ESF: Evaluation of Stress Factors

	Baseline frequency	Post-frequency	P-value	Baseline severity	Post-severity	P-value
Performance in examinations	4.14	4.14	1.0	7.54	7.21	.32
Academic curriculum	3.54	3.29	.18	6.04	5.29	.055
Lack of time for recreation	3.18	3.04	.33	5.07	4.64	.24
Competition with peers	2.75	2.57	.39	4.46	3.93	.21
Performance in practicals	3.18	3.29	.42	5.50	5.93	.26
Loneliness	2.86	2.32	.007*	4.50	3.57	.09
Relationships with the opposite sex	2.86	2.39	.01*	4.64	3.79	.08
Difficulty reading textbooks	2.39	2.18	.18	3.21	2.46	.02*
Adjustment with roommate(s)	2.18	2.04	.33	3.21	3.04	.60
Sleeping difficulty	2.71	2.32	.03*	4.46	3.79	.04*
Exercise	2.82	2.39	.02*	4.36	3.50	.03*
Alcohol/drug abuse/smoking	1.39	1.46	.63	1.89	1.89	1.0
Total scale	34.00	31.43	.001*	54.89	49.04	.014*

## Discussion

Participation in the M&H course was shown to have significantly improved medical students’ perceived stress, depression, and stress factor severity and frequency. These results suggest that EAAT may improve these aspects of medical students’ mental health. The proposal of using EAAT for student wellness is not entirely novel. Efficacy has been shown in other areas including the improvement of mindfulness in medical students, although this study focused specifically on areas of known high risk, namely stress and depression [15].

Based on these findings, the integration of this course into the medical school curriculum would be beneficial as a method of decreasing stress and depression and could be a viable option for students to improve their mental health while also practicing transferrable communication and teamwork skills. Future studies should focus on enlarging the pool of medical student participants with the addition of a control group to obtain more robust data. Additionally, future studies would benefit from the use of physiologic measures, such as salivary cortisol levels or changes in heart rate variability. 

While results from this study are promising, the small sample size limits any ability to generalize the results of this study to the overall population of medical students. The absence of a control group may also limit the ability to compare and apply the results to other groups of medical students. The subjective nature of self-report surveys and the potential for human error or misinterpretation may limit the accuracy of the results, although the use of validated instruments mitigates this potential error. Additionally, data regarding extraneous variables, like life events such as family strain, were not collected; and some scale items may have implied a gender bias (e.g., relationships with the opposite sex), and these could affect the responses received and limit the accuracy of the results. Despite these limitations, the decreases in depression levels were notable, given the short intervention time. At baseline, seven participants scored in the clinical range for depression, while only three had clinically relevant scores after seven sessions. On the PSS scale, only 12 participants (43%) had scores above the midpoint at baseline, and half of those dropped below that point at post-assessment. Stress and depression are significant risks among medical students, putting them at increased risk for long-term chronic health concerns and suicide. If a brief active module that addresses this issue could be added to the medical school curriculum, there might be an opportunity to improve communication and teamwork skills while alleviating depression and the impact of stress factors.

## Conclusions

It is estimated that the levels of stress and depression among medical students are two-five times higher than those in the general population. Participation in the M&H course showed significant improvement in perceived stress, depression, and stress factor severity and frequency for medical students in our study. Integration of this course into the medical school curriculum would be beneficial as a method of decreasing stress and depression while concurrently providing opportunities to practice communication and teamwork skills to bolster both patient and colleague relationships. Future studies should be focused on enlarging the pool of medical student participants to obtain more data.

## Appendices

**Table 2:** Example activities in the M&H course M&H: Medicine and Horsemanship

Session	Activity
Session 1	Round up/turn out – three horses are loose in the area and the students observe the horse behavior and communication from outside the area. Discussion engages the perception of the effects of each horse’s behavior on the dynamic of the herd.
Session 4	Am I being herd? – three students are assigned per horse. Group is instructed to enter the arena and work together to turn the horse in both a clockwise and counterclockwise direction without using any halters or lead ropes.
Session 5	Blindfold obstacle course – a group of two students and one horse navigates an obstacle course in the arena. One student is blindfolded and leading the horse and the other student can see and uses verbal commands to instruct the blindfolded student and horse through the course.
Session 7	Rotating extended appendages – one horse is assigned per three-student team. Students link arms. The student on each end can use their one free arm but cannot speak. The student in the middle can only speak and cannot use either arm. The team of students is then required to place and cinch a bareback pad securely on a horse.
